# Cases and Observations Illustrating the Application of Moxa to the Treatment of Chronic Affections of the Limbs

**Published:** 1827-10

**Authors:** J. Boyle

**Affiliations:** Surgeon to the Middlesex Infirmary.


### MOXA.

### *Cases and Observations illustrating the Application of Moxa to the Treatment of Chronic Affections of the Limbs.* By J. Boyle, Esq. Surgeon to the Middlesex Infirmary.

Case I. *Peculiar affection of the Leg, of upwards of two years' standing, cured by Moxa.*

Mrs. Herbert was admitted a patient of the Middlesex Infirmary on the 10th of December, 1825, for the treatment of a painful affection of the anterior part of the left leg, of (then) about fourteen months' standing. On examination, a very slight enlargement, occupying a space of four inches longitudinally, and about one transversely, commencing two inches below the tubercle of the tibia, was observable; and the skin exhibited a rather dark or livid appearance. The pain was not constantly regular in intensity, but commencing at irregular periods, first in the centre of the part described, and occasionally quickly shooting to the back of the leg. The parts were not uniformly hot, nor was there that peculiar throbbing sensation which ordinarily accompanies inflammation; yet, it having been supposed that this state in its chronic form existed, leeches and cold applications were directed to be applied. These had the effect of lessening, in a slight degree, the frequency of the painful paroxysms; but a repetition of them (the leeches) soon after appeared necessary, and then their employment was not attended with the same advantage in regard to the diminution of pain.

Camphorated mercurial ointment was ordered to be rubbed over the part on going to bed, a flannel bandage being afterwards applied; and the bowels were acted on by alterative medicine. This practice having no effect after a continuance of several days, the tartarised antimonial ointment was next tried, with the same result. The administration of colchicum, and the repeated application of blisters, were not more successful. Moxa, according to the mild mode of applying it generally adopted by me, was productive of but temporary advantage. Hot fomentations with poppies, and hot poultices, produced no effect whatever; and cold applications caused a great increase of pain.

The patient's general health now became gradually affected, and her strength and appetite suffered considerably. Bark was administered, and had the effect of improving her general health, but without in the slightest degree producing any favourable change upon the local disorder.

Alterative doses of calomel, with antimony and opium, were next given, resuming the use of the camphorated mercurial ointment, till slight soreness of the mouth was established. This had the effect of lessening the pain, but did not remove it; and was of temporary service only, as the remedy could not be continued with safety to the general health. The pain soon again returned, with some slight degree of increased heat and a throbbing sensation. Leeches were applied, and had the effect of stopping the inflammatory symptoms. Sarsaparilla powder and nitric acid were now administered, and for a few days the symptoms were less severe than they had been: they again, however, returned, and leeches were again twice applied, with similar palliative effects only.

Thickening of the integuments covering the shin-bone, to the extent of some inches, gradually took place. The pain at this period became much more severe, and was described as if centered in the bone. The sleep was greatly disturbed, and the appetite and general health were both much affected. Supposing now that periosteal inflammation existed, I proposed dividing the integuments as far as the diseased action in them appeared to extend. This was accordingly done, exposing the bone to the extent of about five inches: the integuments were found to be so altered in structure as almost to resemble the thickest leather, but the periosteum and bone appeared perfectly healthy. It was hoped, however, that mitigation of pain would follow the discharge consequent on the formation of the wound, or the probable division of some nervous twigs running through the diseased integuments: and, for the first few days, it did appear that good had been done by the operation. The wound, therefore, was kept open by savine ointment, and a soft light poultice was placed over all, for the purpose of favouring a discharge. The pain again, however, returned, and but little, if any, benefit was derived.

The carbonate of iron was now given, in at first half-drachm doses, and afterwards in drachm doses, every six hours. After giving this a fair trial, it proved entirely useless; the pain being, if any thing, more severe, whilst the appetite and general health were now both greatly impaired, from the intensity of the local disorder.

On the 4th of July, the pain had become almost incessant, commencing in a lateral direction to the wound internally, extending to the foot, and being most violent in the ankle-joint. The poultices were left off; the wound was dressed simply, in order to favour the healing process; and a belladonna plaster was placed over the joint, extending from the internal to the external malleolus. Carbonate of soda, and powdered Peruvian bark, of each seven grains, to be given every two hours, instead of the iron. This last, being continued several days without any advantage, I requested Dr. Johnson to see the patient; the result of which was a coincidence of sentiment in favour of a caustic issue, which was accordingly made in the course of the most painful part, and a double line of peas was introduced; but this was productive of so much uneasiness that, at the urgent request of the patient, I was obliged to heal it up again. After this, spirit lotions with opium, compound camphor, liniment with opium, iodine ointment, and the internal use of iodine, were successively, but unsuccessfully, had recourse to; the pains becoming gradually more severe, sickness of stomach, the greatest degree of general emaciation, and occasional convulsive fits, apparently attributable to the excruciating pain in the leg, threatened a speedy termination of suffering.

Being now nearly exhausted in regard to remedies, I urged the expediency of again trying moxa, but with a greater degree of intensity than that practised on a former occasion. The proposal having been acceded to, on the 28th of October, one moxa cylinder was burnt over the most painful part, occasionally, but not always, in actual contact with the skin, the lighted end being uppermost. A soft light poultice was placed over the part, and the patient enjoyed what she had long been a stranger to—a sound night's sleep: she had no pain for a week after, and then it was comparatively slight. Apprehending its return, however, another moxa cylinder was applied in the manner described; since which all pain has been absent, the appetite is improved, and the patient's strength is already greatly recruited.

In addition to the medical gentlemen whom I have already named, Mr. Jewel, Mr. Bennet, and several others, have seen the subject of this case.

I would only further add, that the painful sensations which have been described, and which were stated by the patient to "dart like lightning" from the point at which they commenced to the toes, never appeared to originate in any part of the anterior tibial nerve. I think it right to state this, lest it might be supposed that the fact was otherwise, and that the early division of that nerve might have prevented much subsequent suffering.

*Case* II. *Contracted Knee-joint, of sixteen years' standing, cured by Moxa.*

On the 19th of October last, Sarah Hubbart, ætat. thirty-two, (No. 12, Swallow-street, Piccadilly,) was admitted a patient of the Middlesex Infirmary. The history of this case was, that, when the patient was sixteen years' old, pain, weakness, and slight swelling were experienced in the right knee, without any apparent cause. A blister was applied, and had the effect of lessening the swelling, but was afterwards followed by a gradual increase of stiffness in the joint and contraction of the limb. At the expiration of seven years from the commencement of the complaint, an accident brought on inflammation, for which she was admitted a patient into one of the public hospitals: here cupping, leeching, and cold applications were practised till the reduction of the inflammatory symptoms was effected, when eleven successive blisters were employed; issues were also had recourse to during her sojourn of seventeen months, of which sixteen were spent in bed, in the hospital; but all to little purpose, except freedom from pain; for, instead of the limb being at an obtuse angle, as was the case on entering upon the above treatment, its extremities had at this time, as nearly as possible, formed a right angle.

All hopes of amendment were now given up by the patient, and nothing further was attempted to better her condition, till about six months previous to the date of her entry on the Infirmary-book; when, from a fall, to which inflammatory symptoms succeeded, she was again obliged to seek medical aid, through which and the usual means the inflammation was soon reduced; and the practitioner in attendance recommended and introduced a seton; which, however, was equally unavailing with the remedies noted above.

On application to me, the knee was considerably enlarged and misshapen, and the integuments, from much exposure to external applications, appeared to be inseparable from the indurated parts to which they gave covering; the limb, as before stated, was at a right angle; the flexor tendons were extremely rigid; the patella had motion, but rested on the outer condyle of the femur, considerably out of its natural situation; and, on either side of its inferior apex, considerable thickening and condensation of what was presumed to be cellular membrane involved the neighbouring portion of the ligamentum patellæ. There was no pain unless violence was used; and, if any inflammation then existed, it must have been very deep-seated, and extremely chronic in its character. A slight degree of flexion could be effected by the voluntary powers of the muscles of the limb, but extension beyond the angle described appeared not to be within the manual efforts of a second person. The daily employment of moxa, according to my usual mode of applying that remedy, was forthwith commenced; a genu-rector, such as I have before spoken of in my published cases, was next prepared, and used thrice daily; and another instrument, entirely novel in its plan and simple in its mechanism, was prepared, and employed as quickly as possible for the purpose of keeping up constant action, to be worn day and night, except when the other measures just named were being employed. This latter instrument* consisted of two light iron sides, attached at either end by what is termed a garter, which was neatly padded, one for giving support to and acting high up on the thigh, the other for the purpose of acting on the os calcis, and thus forming a long and powerful lever; while, opposite the articulation of the knee, a flat joint subjected the action of all the lower part of the instrument to the constant extending power of a spiral spring, so constructed as to press forward at a force equal to fifty pounds; all being kept in apposition by means of a knee-cap. To prevent the instrument from falling off, a light iron stem, with a flat joint opposite the hip, ascended as far as the waist, and was there secured by an elastic belt.

In a few days from the commencement of the means described, increased motion, as well as diminished size of the knee-joint, and considerable relaxation of the integuments covering it, were the consequence. This improvement gradually increased for the first fortnight, at the end of which time, from over-zeal to effect a rapid cure, swelling and inflammation, requiring rest and the application of leeches and evaporating lotions, took place. This state being in a few days removed, small doses of digitalis and greater caution were resorted to, in order to prevent its return. The same treatment was strictly and daily persevered in, and, at the expiration of three months from the patient's entering upon the above means of cure, the limb admitted of being brought perfectly straight under the genu-rector; the knee and leg had acquired a natural and healthy appearance, and, on standing erect, the heel came in perfect contact with the ground.

I would only further observe, that this case has been seen in different stages of its progress by a great number of the profession.

* Manufactured by Mr. Sheldrake, of the Strand.

*Case* III. *Contracted Knee-joint, of five years' standing.*

On the 25th of December last, I was consulted on the case of Miss D—, a child of seven years old, who had laboured five years under a stiff and contracted knee-joint. This case, like the preceding, commenced without any apparent cause; but, different from it, every advantage likely to accrue from private advice was early and zealously sought after in this. The child went on crutches, the leg being nearly at right angles and projecting behind; the knee was enlarged, whilst the muscles of the leg and thigh were flabby, small, and ill-defined.

The same measures as those described in the preceding case,—namely, the application of moxa, the use of the genu-rector, and that also of the more permanent instrument already described,—were employed; the crutches were in a few days laid aside; and, to prevent any unnecessary recapitulation of words, a daily improvement, as in the other case, followed, and, in the latter end of February, all further attendance was deemed unnecessary: the limb was now perfectly straight under the genu-rector, whilst, in walking about, there was scarcely any appearance of lameness, and that from weakness only; as, at the above date, the patient was enabled to bring the heel in perfect contact with the ground.

This case also has been seen, in its different stages, by a number of medical gentlemen, who can bear testimony to the accuracy of the above description of it.

*March 10th,* 1827.

